# Nano-thin amorphous tantalum-coated prosthesis for acetabular bone defect reconstruction: *in vivo* study and case series

**DOI:** 10.3389/fbioe.2025.1687455

**Published:** 2025-10-21

**Authors:** Chang Chen, Siqi Wu, Ge Chen, Zhong Li, Xiaofei Ma, Fuyou Wang

**Affiliations:** ^1^ Department of Traditional Chinese Medicine Rehabilitation, Jiangbei Branch of The First Hospital Affiliated to Army Medical University (Third Military Medical University), Chongqing, China; ^2^ Department of Rehabilitation, Affiliated Hospital of Southwest Medical University, Luzhou, China; ^3^ Department of Orthopaedics, Affiliated Hospital of Southwest Medical University, Luzhou, China

**Keywords:** nano-thin amorphous Ta-coating, acetabular bone defects, 3D-printed porous TC4 implant, magnetron sputtering (MS), osseointegration (OI), clinical effecacy

## Abstract

**Background:**

The optimal reconstruction method for complex bone defects remains controversial. This study aims to introduce the innovative concepts of nano-thin tantalum coating titanium-based prostheses in treating complex acetabular bone defects.

**Method:**

Ten minipigs were used for *in vivo* osseointegration test. Three types of scaffolds (TC4, pure Ta and Ta-coated TC4) were implanted in the femoral condyles, while Micro CT and histological staining were performed at 3 and 6 months post-operatively. Meanwhile, a prospective observational study was conducted from May 2023 to April 2024 to evaluate the clinical efficacy of TC4-based Ta-coated prostheses. The symptom relief and functional recovery were evaluated by visual analogue scale (VAS) and Harris hip score (HHS). Regular radiological follow-ups were arranged to monitor clinical outcomes.

**Results:**

All three types of scaffolds had satisfactory bone in-growth, while TC4 scaffolds still seemed inferior to pure Ta scaffolds and Ta-coated scaffolds in terms of early-stage bone in-growth rate. Totally 3 patients were enrolled in clinical series, with an average follow-up period of 21.7 months. All patients successfully underwent surgery. At the latest follow-up, all patients exhibited significant improvements in pain symptoms (as assessed by VAS) and HHS scores. No severe complications such as infection, prosthesis loosening, or vascular and nerve injuries were observed in any patient.

**Conclusion:**

*In vivo* experiments confirmed the Ta coating may contribute to enhanced osseointegration and biocompatibility of TC4-based prostheses. Meanwhile, the clinical efficacy of TC4-based Ta-coated prostheses in treating complex acetabular bone defects was satisfactory, suggesting that the tantalum coating significantly enhances osseointegration, thereby effectively improving clinical outcomes.

## 1 Introduction

Personalized designed prostheses exhibit significant potential in the treatment of large and complex periacetabular bone defects (Zhang et al., 2019) typically caused by tumors, trauma, and revision surgeries (Kokubo et al., 2016; Wang et al., 2024). The hip joint, as the largest weight-bearing joint, necessitates the precise reconstruction of the anatomical structure to maintain its function (Barrientos-Ruiz et al., 2017). Total hip arthroplasty (THA) is regarded as the gold standard for functional reconstruction of the hip joint, where the reconstruction of bone defects plays a pivotal role in enhancing the initial stability and reconstruct the rotational center (Johnston et al., 2025), serving as a vital guarantee of the long-term survival rate of implants (Bus et al., 2017). However, traditional surgical methods, including autologous/allogeneic bone grafting, saddle endoprosthesis (Danışman et al., 2016), ice-cream cone endoprosthesis (Ogura et al., 2018), and modular prosthesis, each presented their own limitations, falling short of meeting the needs for repairing large and complex periacetabular bone defect. Autografting, long been considered the “gold standard” for bone defect reconstruction, is limited by insufficient supply and donor-site complications (Zheng et al., 2020). While allograft bone is not restricted in terms of availability, its high cost, risk of rejection, and potential for disease transmission still constrain its clinical application (Cheng et al., 2022; Han et al., 2019).

**FIGURE 1:**
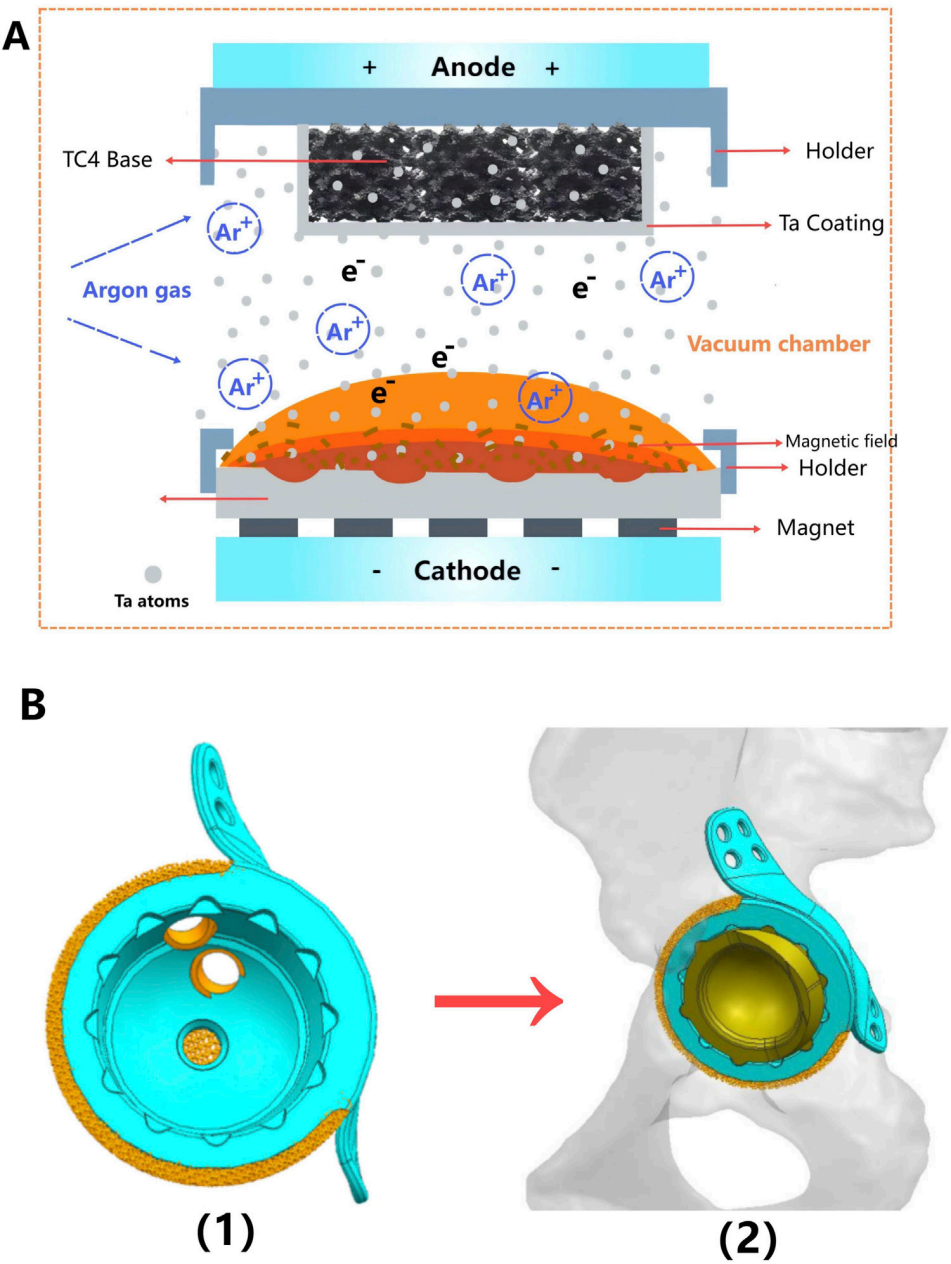
Schematic diagram of preparing a tantalum (Ta) coating on a TC4 substrate and locking structure for the polyethylene liner. **(A)** Schematic diagram illustrating the principle of magnetron sputtering of Ta atoms onto a 3D-printed TC4 substrate. **(B)** The locking structure is machined into the acetabular cup of the monolithic prosthesis using a Computer Numerical Control (CNC) machine, thereby securing the polyethylene liner.

With the development of three-dimensional printing (3DP) technology, personalized designed prosthesis has become a widely applied method for reconstruction of massive acetabular bone defects (Hu et al., 2024). However, the ideal materials for implant are still controversial (Jin et al., 2024). Long-term weight-bearing and stress loading require that those implant not only have better biomechanical strength, but also have enough superior osteogenic properties to avoid the complications including prosthesis loosening or even failure (Brown et al., 2006). Titanium (Ti) and its alloys (Ti-6Al-4V, TC4) are widely used metal materials in bone defect prosthesis due to their excellent mechanical properties, durability and availability (Milivojevic et al., 2023; Guo et al., 2019). However, the relatively poor bone integration ability could be a significant disadvantage for long-term stability of the TC4 implant (Chen et al., 2020; Ding et al., 2024), which may be devastating especially for complex hip reconstruction surgery. Tantalum (TA) is a recognized “biophilic” metal material, which is famous for its superior biocompatibility and osteoinductive properties (Wang et al., 2019; Li et al., 2020). By using customized 3D printed porous Ta prostheses, we successfully reconstructed critical size bone defects in the pelvis and wrist, accurately matching the irregular shape of those defects (Chen et al., 2021; Wang et al., 2024). Meanwhile, the porous structure enabled these prostheses to better integrate with bone tissue. Unfortunately, the scarcity of natural resources has limited the availability of TA and pushed up the production cost, making it economically unable to widely applied in orthopedic implants. To overcome these limitations, our team applied magnetron sputtering (MSP) technology to deposit nano amorphous Ta coating on TC4 substrate (Figure 1A) (Xiao et al., 2021), maintaining the biocompatibility and osteoinductive properties of Ta with the low cost and mechanical properties of TC4.

Most bone defect prostheses were implanted in combination with the traditional THA acetabular cup (Hitz et al., 2023). Different types of materials, fixation with screws and bone cement may lead to the displacement of various components, or even loosening and failure (Chae et al., 2019). Thus, another innovation of this study was that we directly fabricated the integrated hip prosthesis using 3D printing, while manufacturing the locking structure of polyethylene liner in the acetabular cup using computer numerical control (CNC) (Figure 1B). We hypothesized that as Ta coating further optimizing the osteogenic characteristics, integrated prosthesis could achieve a better long-term clinical efficacy.

Through a series of *in vivo* and *in vitro* experiments, our previous studies have effectively proved the safety of the nano thin Ta coating prepared by MSP and the excellent bone integration ability in the early stage of implantation (Wang et al., 2024). This study further conducted *in vivo* experiments of medium and long-term osseointegration evaluation of TC4-based Ta-coated scaffolds. Meanwhile, clinical case series were presented as a preliminary feasibility observation to introduce the complete fabrication process and clinical efficacy of TC4-based Ta-coated prostheses in the treatment of complex periacetabular bone defects.

## 2 Methods

### 2.1 Evaluation of osteogenic properties

Based on the previous study, we selected ten 8-month-old healthy Guizhou minipigs (average weight 35–40 kg) to evaluate the long-term osteogenic ability. The animal ethics committee of the army medical university (AMU) approved all animal experiments. All methods were reported in accordance with ARRIVE guidelines (https://arriveguidelines.org) for the reporting of animal experiments. Commercial spherical TC4 (Ti-6Al-4V) and tantalum (TA) powders with an average diameter of 60 μm were selected to print prosthesis samples (porous cylindrical scaffold, Φ 6 mm × 6 mm, porosity 80%). A single hind limb of each pig was implanted with samples to ensure their normal mobility and symptom control after operation. Two scaffolds were randomly implanted into the medial and lateral condyles of the femur in a single hind limb, ensuring a relatively independent osteogenic environment for each scaffold to avoid mutual interference. All minipigs were assigned unique numbers to facilitate systematic sampling, ensuring that scaffolds of different materials could be evenly collected for subsequent analysis.

After inhalation anesthesia using 3% isoflurane was satisfactory, the hind limbs of the minipigs were shaved, followed by routinely disinfected and draped. Then the skin and muscle were carefully cut layer by layer to fully expose the medial and lateral condyles of the femur. Then, cylindrical bone defects (Φ 6 mm × 6 mm) were drilled. Then, the sterilized TC4 scaffolds, Ta-coated TC4 scaffolds and pure TA scaffolds were randomly implanted. After confirming the stable implantation, the incisions were sutured layer by layer. In total, 7 TC4 prostheses, 7 Ta-coated TC4 scaffolds, and 6 pure Ta scaffolds were implanted across all experimental animals. During the first week after the operation, each minipig was intramuscularly injected with penicillin and meloxicam every day. Minipigs were euthanized after inhalation anesthesia with 3% isoflurane at 3 months (5 pigs) and 6 months (5 pigs) after operation, and the samples with surrounding bone tissue were taken out for Micro CT and hard tissue staining section observation. Since the Micro-CT images of the Ta samples had severe artifacts and could not show the true bone ingrowth, only the TC4 samples and Ta-coated TC4 samples were examined. CTAn v1.19.4.0 software (Bruker, Belgium) was used for quantitative analysis of Micro-CT data. A Region of Interest (ROI) was selected within the scaffold area, while the ROI range covered the entire porous scaffold as well as the newly formed bone tissue within 2 mm around the scaffold, avoiding the inclusion of original bone tissue far from the scaffold. The following key osteogenic indicators, including Bone Volume/Total Volume (BV/TV, %), Bone-Implant Contact (BIC, %) and Trabecular Thickness (Tb.Th, μm) were calculated. Statistical analysis was performed using SPSS 26.0 software, with measurement data expressed as mean ± standard deviation (SD). Between-group comparisons were conducted using the independent samples t-test, while intra-group comparisons at different time points employed paired samples t-tests. The significance level was set at α = 0.05. For each sample, 3 parallel sections (upper, middle, and lower layers perpendicular to the scaffold axis, with an interval of 1 mm) were selected repeatedly for ROI drawing and indicator calculation. The average value of the 3 measurements was taken as the final quantitative result of the sample to reduce measurement errors.

Specifically, at the 3-month follow-up, a total of 3 TC4 prostheses, 4 Ta-coated TC4 scaffolds, and 3 pure Ta scaffolds were harvested from the euthanized pigs. At the 6-month follow-up, 4 TC4 prostheses, 3 Ta-coated TC4 scaffolds, and 3 pure Ta scaffolds were obtained from the 5 euthanized miniature pigs for subsequent experimental evaluations.

### 2.2 Case series

A prospective observational study was conducted to assess the clinical outcomes of utilizing a 3D-printed Ti-based Ta-coating prosthesis for the reconstruction of massive acetabular bone defects. From May 2023 to April 2024, three patients (two males and one female) who fulfilled the inclusion criteria took part in our study. Each patient was assigned a unique number for clarity (refer to Table 1). Exclusion criteria encompassed patients requiring radiotherapy, chemotherapy, or long-term corticosteroid treatment; patients with a history of previous hip revision surgery (excluding primary total hip arthroplasty) or hip surgery due to tumors, infections, or other complex etiologies; and patients who were unwilling to undergo surgery or complete follow-up. Informed consent was obtained from all patients.

**TABLE 1:** Baseline statistical data of patients.

Patient	Age	Gender	Side of operation	Position	Cause	Follow-up (m)
1^a^	74	female	Left	hip joint	THA repair	26
2^a^	47	male	right	pelvic	chondrosarcoma	24
3^a^	66	male	right	pelvic	chondrosarcoma	33

^a^ Received blood transfusion therapy after surgery.

In the results section, we will illustrate the entire process of prosthesis fabrication and surgical implantation through a typical case study (Patient 1).

#### 2.2.1 Image acquisition and prosthesis design

**FIGURE 2:**
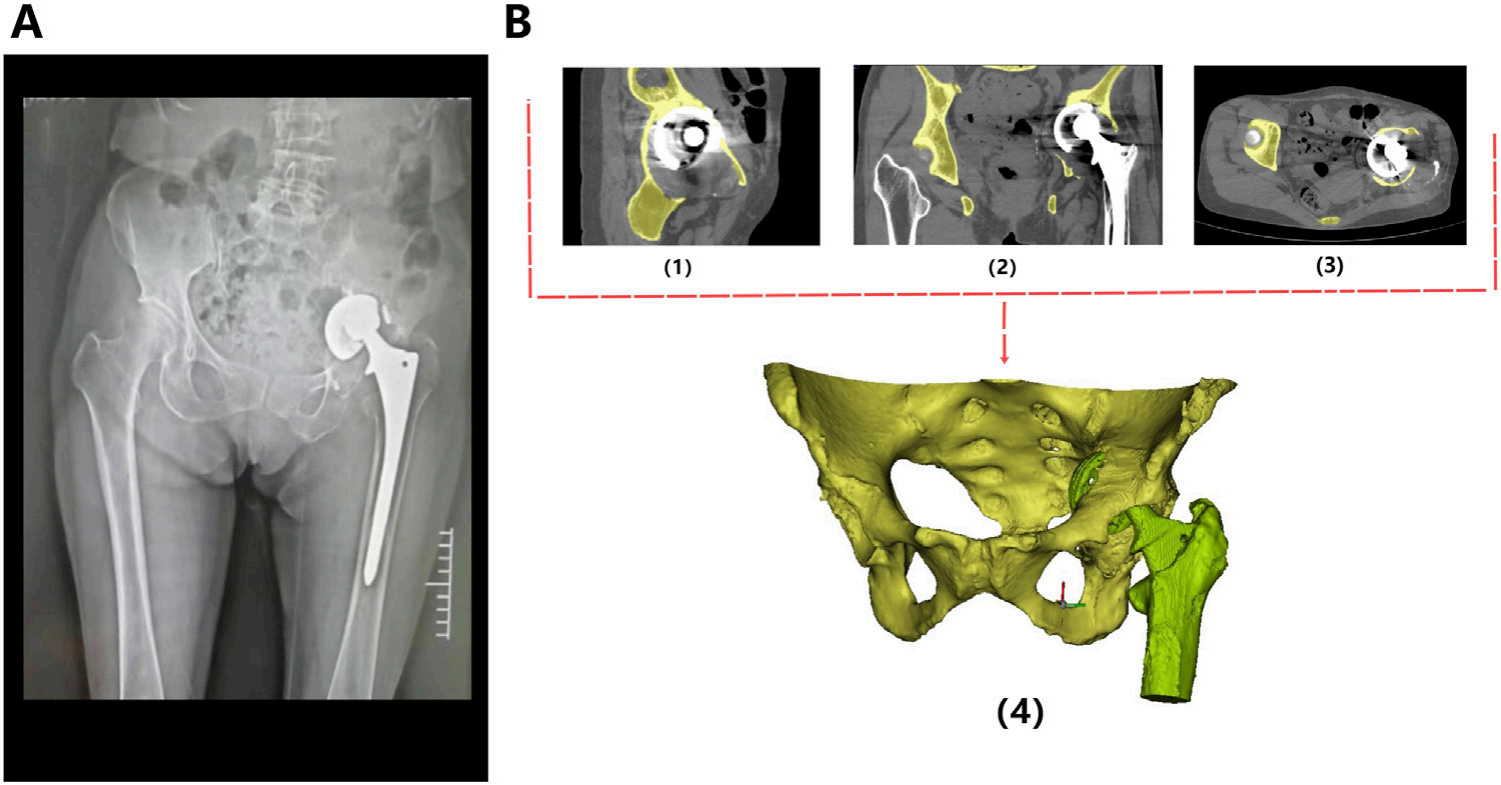
Preoperative radiological image of the patient. **(A)** The X-ray revealed loosening and dislocation of the acetabular cup. **(B)** CT scan images of the bilateral hip joints in sagittal (1), coronal (2), and axial (3) planes. Based on the CT data, a three-dimensional reconstruction was performed using MIMICS software (4), which stereoscopically demonstrates the morphology of the pelvis and proximal femur, as well as the location of the implants.

All patients underwent high-resolution (512 px×512 px) thin-layer computed tomography (CT) with a slice thickness of 1 mm. The scanning area should cover the ipsilateral and contralateral limbs of the bone defect, thereby providing detailed imaging data of adjacent anatomical structures (Figure 2B).

**FIGURE 3:**
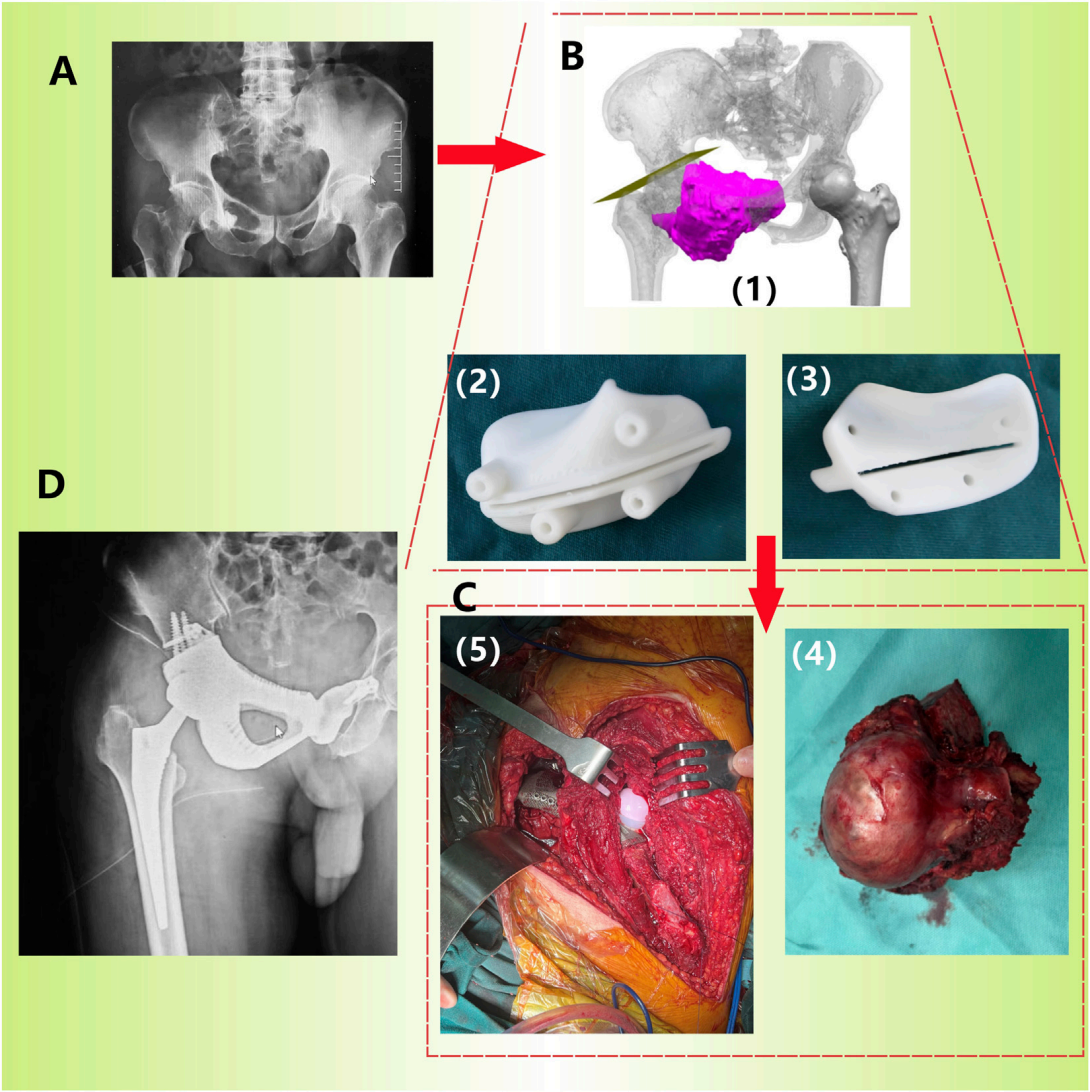
Procedure of prosthetic implantation surgery (Patient 2). **(A)** Preoperative pelvic X-ray images show lesions in the right pelvis; **(B)** Preoperative 3D planning: determination of osteotomy plane (1) and printing of osteotomy guide plate (2/3: different angles); **(C)** Accurate resection of tumors (4) and implantation of prostheses (5) using guide plates during surgery; **(D)** X-ray image taken 1 month after surgery.

The original CT data was converted into the Digital Imaging and Communication in Medicine (DICOM) format and carefully stored in the mobile hard disk. Mimics Innovation Suite 19.0 software (Materialise, Leuven, Belgium) was used for 3D reconstruction of CT images, where the fine adjustment was made according to Hu threshold (bone CT) built in the software to achieve accurate segmentation of tumor and normal bone tissue. Smoothing functions should be avoided as much as possible to minimize distortion and errors in images and data. Then, the processed STL data were imported into Unigraphics NX8 (UG) software (Siemens PLM Software, Texas, United States, URL link: https://plm.sw.siemens.com/en - us/nx/) for 3D modeling, preoperative planning and prosthesis design (Figure 2B). For bone tumors and other cases requiring 3D printing cutting guide plate, the tumor boundary and the osteotomy plane should be determined on the 3D model in advance (Figure 3B1), followed by the final design and printing of the guide plate (Figures 3B2, 3). The attending surgeons should repeatedly communicate with the designer for the most appropriate surgical plan and prosthesis design according to the individual situation of each patient.

#### 2.2.2 Preparation of titanium alloy (TC4) substrate

**FIGURE 4:**
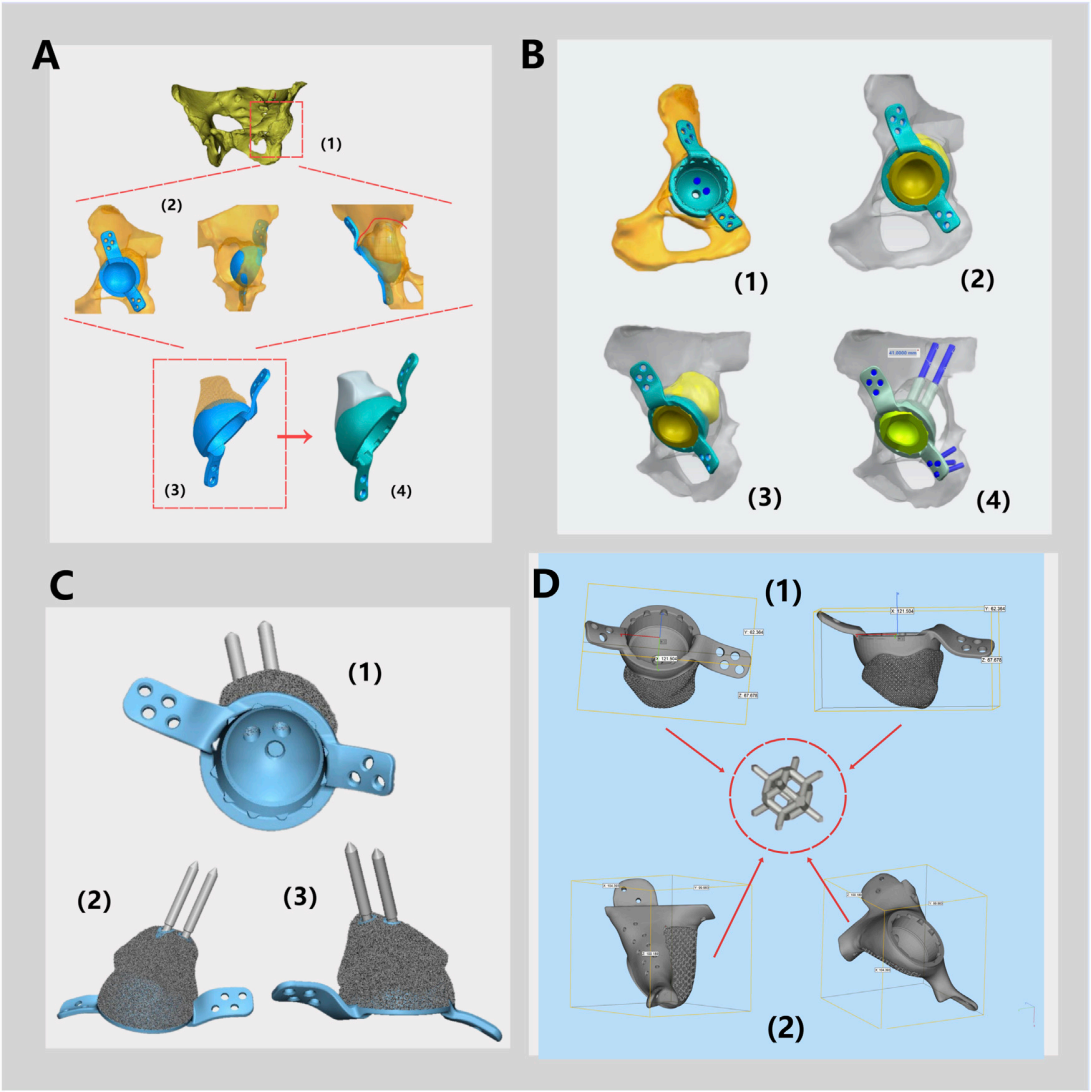
Schematic Diagram of a Monolithic Prosthesis Design. **(A)** Three-dimensional modeling of the pelvis and hip joint (1). According to the bone defects, the placement of the acetabular cup and the shape of the prosthesis for bone defects were simulated (2) to determine the preliminary structure of the integrated prosthesis (3), and the polyethylene liner locking mechanism is designed (4). **(B)** The fixation method for the prosthesis during surgery and the type of screws are determined. **(C)** The prosthesis, featuring fixation pins and polyethylene liner locking mechanism are displayed from various angles. **(D)** General dimensions of prosthesis design of Patient 1 (1) and Patient 2 (2) and schematic diagram of rhombic dodecahedral structure.

Dodecahedral crystal cell structure was determined as the internal porous structure of the 3D printing prosthesis, with a pore size of 600 μm and a porosity of 80% (Figure 4). The substrate was fabricated by using the electron beam selective melting (EB-PBF) system (Sailong Y-150, operating at 60 kV), with high quality commercial spherical TC4 (Ti-6Al-4V) powder with an average particle size of 60 μm. Once the prosthesis was fabricated, high-pressure air was used to completely remove any residual TC4 powder, followed by using anhydrous alcohol and ultra pure water for ultrasonic cleaning for another 5 min.

The printed prosthesis was gradually cooled in a vacuum environment, preventing the risk of deformation and cracking during the cooling process. The acetabular polyethylene liner locking structure was fabricated using a CNC machine (Figure 1B). In this study, the polyethylene liners implanted in all three patients were High Cross-Linked Ultra-High Molecular Weight Polyethylene (HXLPE) liners supplied by Johnson & Johnson MEDICAL (CHINA) Ltd. Notably, the locking structure of the 3D-printed integrated acetabular cup was specifically designed to match the structural features of this company’s HXLPE liners, ensuring precise mechanical fitting between the liner and the acetabular cup to avoid interfacial micro-movement.

#### 2.2.3 Preparation of the nano thin tantalum coating

MSP machine (JGP-560B, Shenyang Scientific Instrument Co., Ltd.) was used to deposit tantalum coating on 3D printed TC4 substrate. Before coating preparation, pre-sputtering process should be performed at 1 Pa argon pressure and 120 W sputtering power for 1 min to eliminate oxides and impurities on the surface of Ta target. The magnetron sputtering power of the coating fabrication was 250 W, with 0.6 PA of the argon pressure and 30 s of the sputtering time (Figure 1A). In order to ensure the completely coating coverage on the porous structure, the TC4 substrate was rotated four times from different directions (up, down, front and back) to achieve the uniform deposition.

#### 2.2.4 Surgical procedures

Prior to surgery, the surgical planning and designed prosthesis must be discussed by the department’s surgeons, including the professor team of the bone tumor and joint surgery professions. Every detail of the surgical process and possible situations need to be discussed and rehearsed.

Throughout the surgery, strict adherence to aseptic techniques is essential. All patients should undergo general anesthesia (1.5–2.5 mg/kg Propofol by intravenous injection and 3% Sevoflurane by inhalation). Sufficient blood transfusion must be prepared before surgery because of the significant trauma. Both the lateral decubitus position and the floating position can be chosen as the surgical position as long as the surgical incision can fully exposes the surgical area. During the operation, it is essential to avoid unnecessary damage to blood vessels, nerves, and soft tissues. A preoperative designed 3D osteotomy guide plate can ensure precise osteotomy positioning, enhancing surgical efficiency (Figure 3). Meanwhile, repeated C-arm fluoroscopy is necessary to confirm the satisfactory position of the implantation. Hemostasis must be thoroughly performed to prevent postoperative hematoma formation.

#### 2.2.5 Postoperative management

Postoperatively, all patients should stay in safe posture (Toyoda et al., 2023) to prevent prosthesis dislocation, avoiding adduction, internal rotation, and excessive flexion (<90°) of hip. Intravenous controllable analgesia pumps were applied to all patients, combined with standardized multimodal analgesia including intravenous or oral administration of NSAIDs, tramadol, hydrocodone or topical buprenorphine, etc. Within 48 h after surgery, ice bags were applied to reduce swelling. Additional nerve block may be given to those patients with severe pain symptoms (VAS ≥5). Regular antibiotics should be given daily for 14 days postoperatively to prevent infection. Daily anticoagulation with low molecular weight heparin sodium should be performed from 12 h after surgery. After discharge, oral rivaroxaban was given until 5 weeks after surgery to prevent thrombosis.

On the first day after surgery, active training including ankle pump and quadriceps isometric contraction should be performed under the guidance of rehabilitation therapists. Four weeks postoperatively, active hip training could be carried out, while excessive activity should be avoided. Weight-bearing and strenuous activities should be prohibited on the affected limb within 3 months after surgery. Patients were evaluated clinically using the Harris hip score (HHS) and radiologically using postoperative X-rays. Regular follow-up is done in all cases at the first, third, sixth, and 12th months. Any complications related to implants and wounds will be recorded.

## 3 Results

### 3.1 Evaluation of osteogenic properties

**FIGURE 5:**
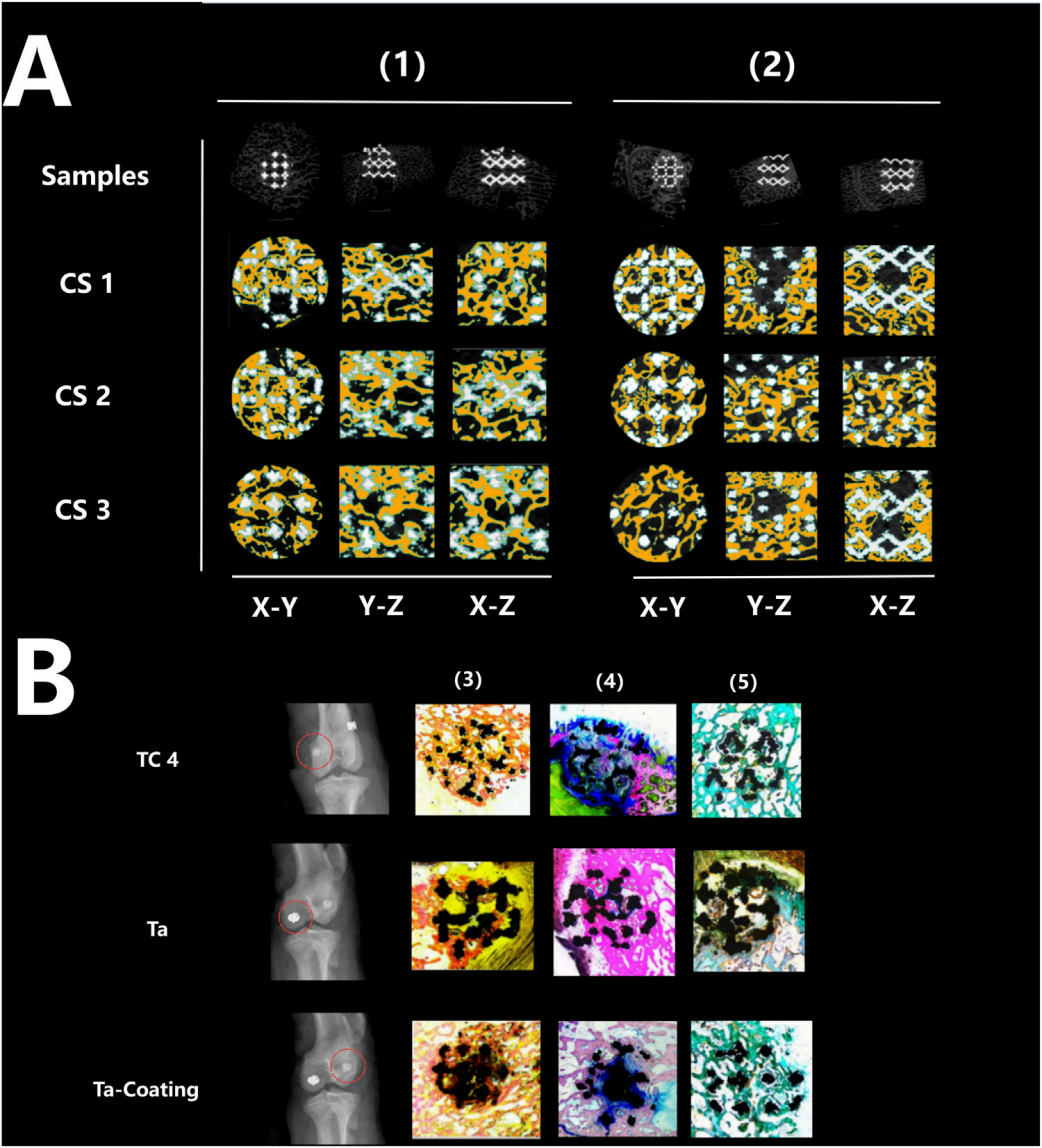
Micro-CT imaging and histological staining 3 months after implantation. **(A)** Micro-CT results: the samples of TC4 scaffolds (1) and Ta-coated scaffolds (2) were not displaced, and new bone tissue (yellow) grew around and inside the two groups of scaffolds from different cutting directions and planes. **(B)** Histological staining results: X-ray images showed that the three groups of scaffolds (TC4, Ta and Ta-coating) were fixed without displacement (red circle), and the representative images of Sirius red staining (4), Goldner staining (5) and methylene blue acid magenta staining (3) showed that the bone in-growth in each group, reaching the center of the scaffolds, and the fibers were evenly distributed.

**FIGURE 6:**
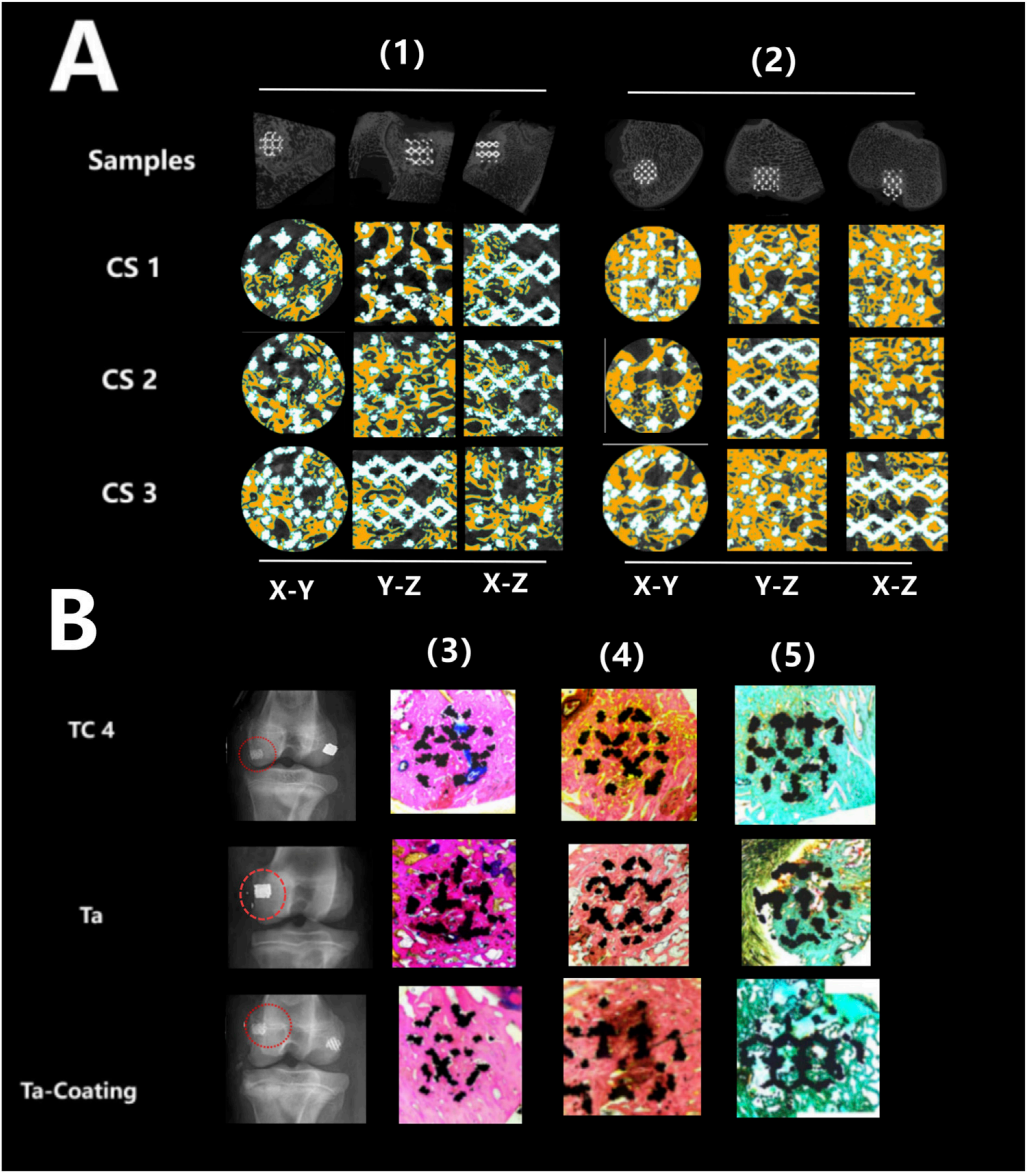
Micro-CT imaging and histological staining 6 months after implantation. **(A)** Micro-CT results: the samples of TC4 scaffolds (1) and Ta-coated scaffolds (2) were not displaced, and new bone tissue (yellow) grew around and inside the two groups of scaffolds from different cutting directions and planes. **(B)** Histological staining results: X-ray images showed that the three groups of scaffolds (TC4, Ta and Ta-coating) were fixed without displacement (red circle), and the representative images of Sirius red staining (4), Goldner staining (5) and methylene blue acid magenta staining (3) showed that the bone in-growth in each group, reaching the center of the scaffolds, and the fibers were evenly distributed.

Figures 5A, 6A showed that all three types of scaffold (TC4, Ta and Ta-coated TC4) in minipigs did not have dislocation, loosening or fracture within 6 months after implantation in the femoral condyle. Since the Micro-CT images of the Ta samples had severe artifacts and could not show the true bone ingrowth, only the results of TC4 samples and Ta-coated TC4 samples were shown in Figures 5A, 6A. In order to better observe the bone in-growth, we selected three planes for observation (X-Y, x-z and Y-Z). Three cross-sectional positions (CS-1, CS-2 and CS-3) parallel to these planes were selected to better observe the trend of osseointegration. At 3 months (Figure 5A), the new bone tissue (yellow) grew well around and inside both groups of scaffolds. Meanwhile, the new bone in-growth density in the Ta-coating group samples (Figure 5A1) was higher than those in TC4 (Figure 5A2). At 6 months, the in-growth density of new bone in the Ta coating group (Figure 6A1) sample was still higher than that in TC4 (Figure 6A2). The quantitative analysis results (Table 2) of Micro CT showed that at 3 and 6 months after surgery, the BV/TV, BIC, and Tb. Th of Ta-coated TC4 scaffolds were significantly higher than those of TC4 stent during the same period (P < 0.05), indicating that the Ta coating can sustainably promote the formation and maturation of new bone around the scaffolds, while this effect was more significant in the early postoperative period (3 months). These results were consistent with our previous *in vivo* findings (6 weeks) (Wang et al., 2024) that the early-stage bone ingrowth rate of the Ta-coated scaffolds was higher than that of the TC4 scaffolds.

**TABLE 2:** Comparison of Micro CT quantitative indicators between TC4 and Ta coated TC4 scaffolds (x ± s).

Micro CT quantitative indicators	TC4 scaffolds	Ta-coated TC4 scaffolds	t value	P value
3 months				
BV/TV (%)	18.2 ± 2.5	31.1 ± 3.9^a^	6.329	<0.05
BIC(%)	32.6 ± 3.8	54.2 ± 5.5^a^	7.014	<0.05
Tb.Th (μm)	48.3 ± 4.2	65.1 ± 6.0^a^	5.842	<0.05
6 months				
BV/TV (%)	25.7 ± 3.1	35.8 ± 4.3^a^	8.156	<0.05
BIC(%)	45.9 ± 4.5	62.7 ± 6.1^a^	9.038	<0.05
Tb.Th (μm)	56.7 ± 5.1	71.2 ± 6.5^a^	7.569	<0.05

^a^ The difference is statistically significant (P < 0.05).

The histological evaluation of bone defects repair and bone formation was carried out by Sirius red staining, Goldner staining and methylene blue-acidic magenta staining. At 3 months (Figure 5B) and 6 months (Figure 6B), Sirius red staining, Goldner staining and methylene blue-acidic magenta staining showed that the bone in-growth were well inside the porous scaffold in each group, reaching to the center of the scaffold, while the fibers were evenly distributed. However, at 3 months, the fiber density of the TC4 scaffolds was significantly lower than that of the Ta scaffolds and Ta-coated scaffolds. Previous research results (Wang et al., 2024) suggested that at the early stage, the bone ingrowth of Ta-coating group was significantly better than that of the other two groups. This study showed that at 3 months after surgery, the bone ingrowth of Ta scaffolds and Ta-coated scaffolds may be better than that of TC4. At 6 months after operation, the difference of bone in-growth between those three scaffolds seemed less obvious.

### 3.2 Clinical results

All three patients, consisting of two males and one female, underwent surgery successfully, as information regarding the clinical histories were provided in Table 1. The surgery duration ranged from 3 to 10 h, averaging 7.3 h. Intraoperative bleeding varied between 600 and 1,500 mL, with a mean volume of 1,200 mL. The hospitalization period lasted 10–14 days, averaging 12.7 days. All patients required blood transfusions. After the surgery, all patients only experienced mild complications such as pain and swelling, which were effectively managed with symptomatic treatment, and no significant discomfort was reported subsequently. All patients’ body temperatures normalized within 1 day after surgery. All incisions healed well and achieved A-level healing standards.

**FIGURE 7:**
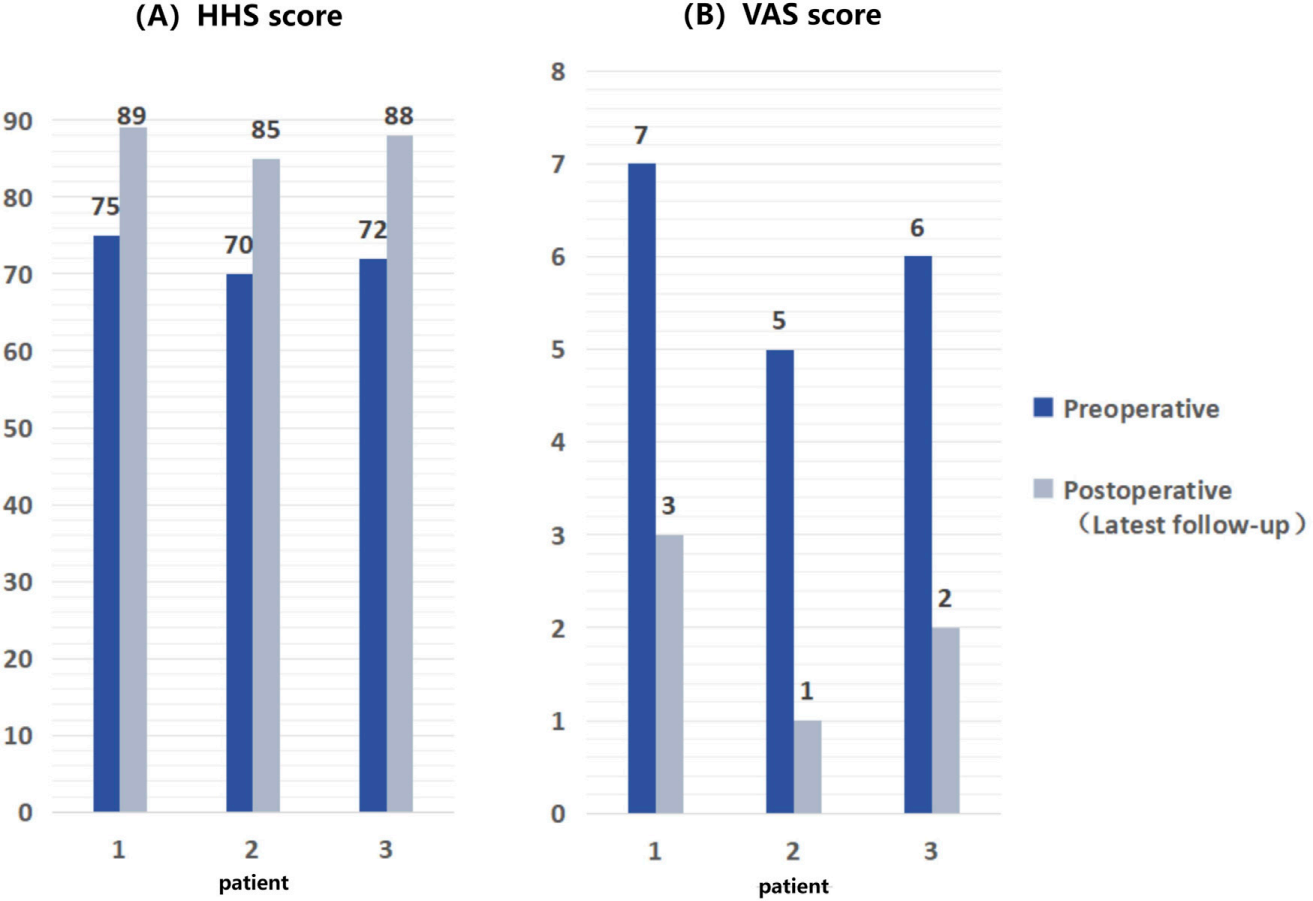
Comparison of HHS scores and VAS scores of patients before and after surgery, showing significant improvement in postoperative scores compared to preoperative scores. **(A)** HHS scores of 3 patients; **(B)** VAS scores.

The follow-up period ranged from 24 to 33 months, averaging 27.7 months. As of their latest follow-up, all patients exhibited significant improvements in pain symptoms (assessed using VAS scores) and HHS scores (as shown in Figure 7). Notably, no patients experienced serious complications such as infection, prosthesis loosening, or vascular and nerve damage.

### 3.3 Typical case - Revision total hip arthroplasty with massive bone defects

Patient 1 is a 74-year-old female who underwent THA 17 years ago due to avascular necrosis of the left femoral head. The current chief complaint was left hip joint pain accompanied by restricted mobility for 4 months. Physical examination revealed a 2-cm shortening of her left lower limb and severe limitation in hip joint movement. X-rays clearly demonstrated signs of loosened and dislocated acetabular cups, along with severe osteoporosis (Figure 2A).

**FIGURE 8:**
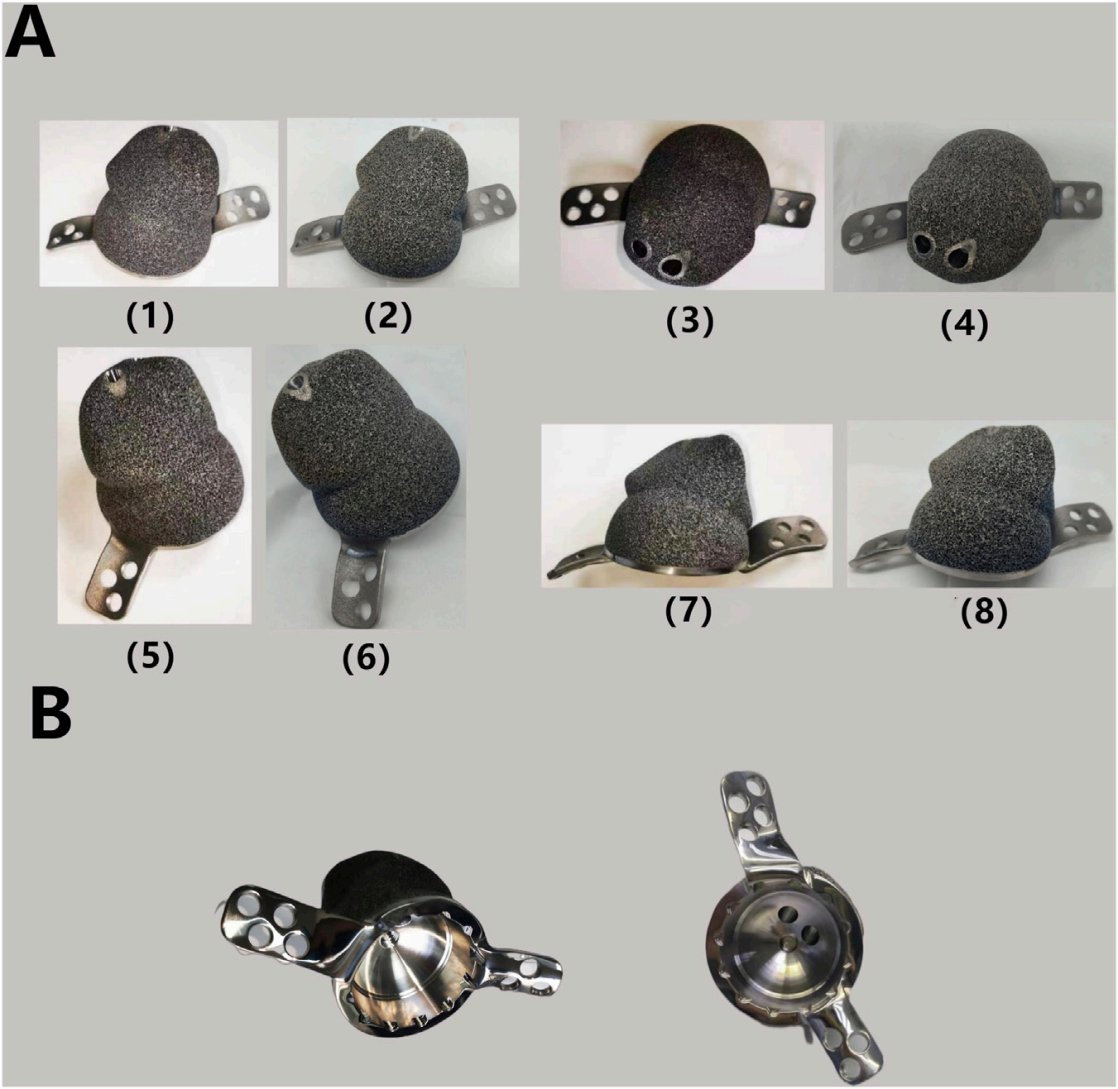
**(A)** Finished TC4-based Ta coating prosthesis: the TC4 substrate after surface polishing (1) (3) (5) (7) and the finished product after Ta coating by MSP (2) (4) (6) (8). The surface of the implant becomes smoother and exhibits a slight light blue hue after the Ta coating process. **(B)** Polyethylene liner locking structure after processing.

After discussion, the revision surgery focused solely on the loosened acetabular cup. Given the patient’s severe osteoporosis, massive bone defects could result from the removal of the existing acetabular cup. The raw data of thin-layer CT scan was putted into Mimics software to reconstruct her pelvis and hip joint (Figure 2B), revealing a massive bone defect in the acetabulum. After removing the prosthesis imaging, we simulated the bone defects and designed an integrated prosthesis to address the bone defects, in conjunction with the acetabular cup prosthesis (Figure 8). Based on the design, an integrated bone defect prosthesis was 3D printed. The bone defect implant above the cup was constructed using porous TC4 as the substrate, and a nano-thick tantalum coating was subsequently applied to the surface via magnetron sputtering (Figure 8A). The interior of the cup was machined into a slot locking structure using a CNC machine, according to the preoperative plan for the polyethylene liner (Figure 8B).

Under general anesthesia, the patient was positioned in a fixed right lateral decubitus position. Following routine disinfection and draping, a curved posterolateral incision approximately 20 cm was made on the left hip. The skin, subcutaneous tissue, and deep fascia were dissected layer by layer, while electrocautery was carefully applied for hemostasis. The gluteus maximus and tensor fascia lata muscles were then gradually released. With the hip flexed and internally rotated, the joint capsule was incised. Metallic debris, reactive synovium, hyperplastic synovium, inflammatory lesions, and scar tissue were thoroughly removed and sent for bacteriological culture. The wound was thoroughly rinsed with povidone-iodine and normal saline. The femoral prosthesis was exposed and confirmed to be stable without loosening. Then the acetabulum was fully exposed. Hyperplastic synovium, osteophytes, degeneration tissue, and bone within the joint cavity were completely cleaned up. Loosening of the acetabular cup margin was confirmed, prompting the removal of the acetabular cup prosthesis and polyethylene liner. The acetabular was then prepared for the placement of the customized 3D-printed prosthesis. Eight acetabular screws were inserted as the preoperative plan to fix the prosthesis, with C-arm fluoroscopy confirming satisfactory positioning. After repeated rinsing of the joint cavity with povidone-iodine and normal saline, a new polyethylene liner (36 mm polyethylene lining, OD 56 mm, Johnson & Johnson MEDICAL (CHINA) Ltd.) was installed. The prosthesis’ position and stability were verified. The wound was then soaked in povidone-iodine and rinsed repeatedly with pulsed lavage before a plasma drainage tube was placed. The articular capsule was repaired, and the wound was sutured layer by layer, followed by sterile dressing.

**FIGURE 9:**
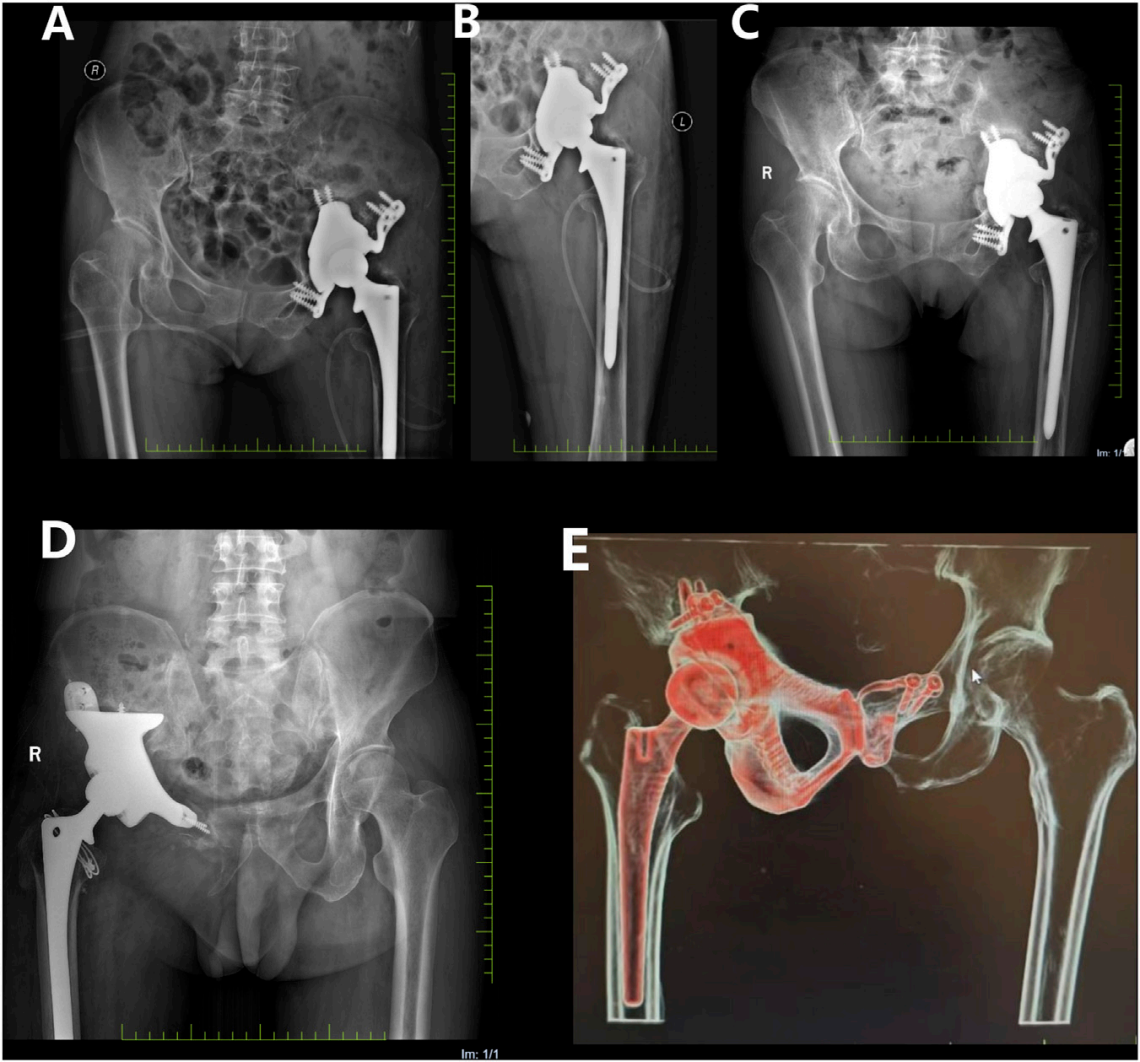
Postoperative X-ray films. **(A,B)** on the first day after operation, the prosthesis position of patient 1 was satisfactory, and there was no sign of displacement or loosening. **(C)** 18 months after operation, compared with the previous X-ray film, the prosthesis position of patient 1 remained satisfactory without displacement; **(D)** At 18 months after operation, the position of prosthesis of patient 3 remained satisfactory without displacement; **(E)** 18 months after operation, the prosthesis position of patient 2 remained satisfactory without displacement.

The postoperative recovery was uneventful, with satisfactory wound healing and suture removal 2 weeks later. The affected limb was kept non-weight-bearing for 2 months postoperatively. Antibiotics were administered to prevent infection, while oral and topical analgesics effectively managed pain symptoms. 18 months after surgery, X-ray examination revealed a satisfactory prosthesis position, with no signs of displacement or loosening (Figure 9A‐C). At the latest follow-up (26 months after surgery), the patient reported good functional recovery of the left hip without any significant complication.

In addition to Patient 1, Patients 2 (47 year old male, huge chondrosarcoma of the right pelvis) and 3 (66 year old male, huge chondrosarcoma of the right pelvis) recovered well after the customized prosthesis surgery, without any serious postoperative complications during the follow-up period (Figures 9D,E).

## 4 Discussion

Here we presented a clinical case series on the application of 3D-printed integrated prostheses in patients undergoing total hip arthroplasty (THA) with massive bone defects. The unique innovations prominently included the utilization of magnetron sputtering (MSP) technology to deposit a nano thin tantalum (Ta) coating onto porous TC4 substrates. Combined with *in vivo* histological test, we aspired to provide valuable insights for future research and the references of additive manufacturing (AM) technologies and Ta coatings in treating complex bone defects.

### 4.1 Histological evaluation of *in vivo* osteogenic properties

In previous studies, at the early stage of implantation, tantalum coated scaffolds showed higher cell proliferation rate and bone in-growth rate than pure tantalum and TC4 (Wang et al., 2024), which may be because the oxidation components (TiO2 and TaOx) on the surface of tantalum coating affect the protein and cell interactions between bone tissues (Chae et al., 2019). As it is still controversial whether Ti-6Al-4V alloys demonstrate poor biocompatibility and osseointegration, further improving the osteogenic ability of the prosthesis could have great significance for the long-term outcomes (Konan et al., 2016). In this study, we further conducted the *in vivo* osteogenesis tests in 3-month and 6-month post-operatively. Our results showed that although all three kinds of scaffolds had satisfactory bone in-growth, TC4 scaffolds still seemed inferior to pure Ta scaffolds and Ta-coated scaffolds in terms of early-stage bone in-growth rate. We believed that the application of Ta coating on porous TC4 prosthesis can effectively improve the early stability of prosthesis, which is important and valuable for hip prosthesis that requiring early movement and weight-bearing.

Combined with our previous research (Wang et al., 2024), both *in vitro* and *in vivo* studies have inferred that Ta-coated scaffolds exhibited better cell proliferation and bone in-growth. We suggested that the unique surface physicochemical properties of nanocrystalline amorphous tantalum coating might be the basis for initiating superior osteogenic reactions. First, the surface of Ta coating presented a uniform micro-nano scale rough structure, which could significantly increase area for the cell attachment compared to the smoother TC4 substrate. Meanwhile, the contact angle of the Ta coating was 42.3° ± 2.5°, which indicated a better hydrophilicity compared with TC4 substrate (78.5° ± 3.2°). This property could effectively promote the adsorption and conformational optimization of extracellular matrix proteins such as fibronectin (FN) and laminin (LN), providing “anchoring points” for the early adhesion of osteoblasts (Wang et al., 2012), thereby activating the focal adhesion kinase (FAK) signaling pathway (Gunn et al., 2022) and promoting cell spreading and proliferation. Moreover, the surface oxidation components formed by the Ta coating, such as amorphous TaOx, can affect the interaction between proteins and cells (Garbacz et al., 2010; Cui et al., 2023), accelerating osteoblast differentiation and bone matrix maturation by activating key osteogenic signaling pathways, such as the BMP-2/Smad (Zhang et al., 2020) and the Wnt/β-catenin signaling pathway (Qian et al., 2020).

### 4.2 The advantages of magnetron sputtering and nano thin amorphous Ta-coating

By introducing the TC4-based Ta-coating implants, as well as the design features of the polyethylene liner’s locking structure, we elucidated how these two innovations facilitate the further promotion of personalized prosthesis for THA with complex acetabular bone defects. In terms of clinical outcomes, our primary results, including pain symptoms and functional outcomes (Harris Hip Score, HHS), demonstrated significant improvement in all patients. Notably, no severe complications such as infection, prosthesis loosening, or vascular and nerve injuries were reported during the follow-up period. Our results indicated that TC4-based Ta-coating prostheses enhanced clinical outcomes.

The main purpose of our research on fabricating Ta coatings was to reduce the cost of prosthesis production while preserving the excellent osteogenic properties of Ta. Ta prosthesis have been extensively reported for clinical applications treating various bone defects, albeit mostly in isolated cases (Ao et al., 2022; Wu et al., 2022). In recent years, many researchers have used Ta for surface modification of conventional biomaterials, which has become an important research direction of orthopedic implant (Cao et al., 2021). Those studies aimed to retain the advantages of traditional materials, while further improving their osseointegration performance, ultimately obtaining better clinical outcomes (Du et al., 2022). To overcome the poor osteogenic ability of titanium alloy (TI), Shi et al. fabricated Ta coating on Ti pedicle screw by chemical vapor deposition (CVD) (Shi et al., 2017), confirming that the Ta coating could further promote bone formation and inhibit osteoclast proliferation through *in vitro* and *in vivo* experiments. However, the disadvantages of CVD are also obvious. Traditional CVD requires high reaction temperature (600 °C–1,100 °C), making heat-resistant substrates, such as PEEK, not suitable for this method (Huang et al., 2021). Polyetheretherketone (PEEK) has excellent mechanical strength and biological stability, and has a more appropriate modulus of elasticity than metal materials, thus avoiding the occurrence of stress shielding (He et al., 2021). However, as a bioinert material, the inferior osteogenic ability limits its application (Xin et al., 2023). Pang et al. (Pang et al., 2021) used vacuum evaporation to deposited tantalum pentoxide (TP) coating on the surface of PEEK implants, which improved the surface properties and cell compatibility. However, the coating prepared by vacuum evaporation is mainly “physically attached” to the substrate, with weak adhesion and easy to fall off when subjected to external forces or temperature changes (Garbacz et al., 2010). Meanwhile, for the prosthesis with complex shape, the uneven atomic evaporation rate caused by temperature difference could lead to uneven film thickness (Kiryukhantsev-Korneev et al., 2022). Compared with CVD and vacuum evaporation, MSP can not only prepare the coating at room temperature, but also has better adhesion and uniformity. By using MSP, Hwang et al. (Hwang et al., 2020) prepared Ta coating on the surface of electrospun PLA membrane for guided bone regeneration (GBR), which confirmed the superiority of its bone conductivity.

Our research team fabricated nano thin amorphous tantalum (TA) coating on porous TC4 substrate by using magnetron sputtering (MSP), with a thickness of about 20 nm. The coating achieved excellent interface bonding, high coverage and significant biocompatibility (Wang et al., 2024), with better cell proliferation rate and bone in-growth rate than TC4 in the early stage. In this study, the 3-month *in vivo* experiment still showed that the density of bone tissue in the central area of Ta-coated scaffolds was better than that of TC4 scaffolds, while this difference existed even at 6 months after implantation. The clinical results of this study showed that during an average follow-up period of 27.7 months, all patients using TC4 based Ta coated THA prosthesis had significant improvement in pain symptoms and functional outcomes, without reporting any serious complications. In addition, our TC4 based Ta coated implants can reduce the material manufacturing cost by about 10 times compared with implants using pure Ta, while achieving satisfactory osteogenic ability. We indeed did not conduct specialized mechanical strength tests on the prostheses. This was mainly because the application of porous titanium alloys in bone defect prostheses was already quite widespread, and the manufacturing parameters in this study can also ensure sufficient mechanical support for the prostheses. Meanwhile, as the hip joint is a major weight - bearing part of the human body, the average follow - up period of 27.7 months in this study without any deformation, displacement, or loosening of the prostheses occurred could further verify the excellent mechanical strength of the titanium-based tantalum-coated prostheses. All the results indicated that our innovative TC4 based Ta coated prosthesis can provide more effective treatment options for various bone defects.

### 4.3 Innovations and advantages of the polyethylene liner locking structure

In brief, the major significance of the polyethylene liner locking structure lies in its ability to integrate bone defect augments with the acetabular cup, thereby eliminating the dependence on traditional acetabular cups. Previously, only traditional acetabular cup prosthesis from certain manufacturers featured locking structure for securing polyethylene liners. Therefore, regardless of whether metal augments or other reconstructive techniques were utilized, fixation with the acetabular cup prosthesis was inevitable (Beckmann et al., 2018). Although both conventional metal augments and 3D-printed personalized augments have reported favorable long-term clinical outcomes, the hip joint serves as a primary weight-bearing anatomical structure in the human body, and the risk of long-term failure significantly increases with complex modular prosthesis. In a clinical study by Whitehouse et al. (Whitehouse et al., 2015) on acetabular trabecular metal augments, the 10-year survivorship of the prosthesis was 92% in 53 patients, with three experiencing prosthesis loosening leading to revision surgery. Löchel et al. used tantalum blocks, with a 10-year prosthesis survivorship of 95.2%, although three patients (5.6%) developed loosening and underwent revision surgery (Löchel et al., 2019). Hu et al. employed 3D-printed custom augments and also observed one case of postoperative loosening (Hu et al., 2024). Integrated prosthesis, theoretically, offers the best solution to this problem. Therefore, we have developed an acetabular locking mechanism compatible with polyethylene liners.

Postoperative center of rotation (COR) migration is prevalent in modular prosthesis. Xiao et al. (Xiao et al., 2021) used porous titanium-coated tantalum metal augments for revision total hip arthroplasty (THA), reporting vertical COR migration ranging from 11.7 to 42.9 mm and horizontal migration from 20.8 to 49.2 mm. Additionally, two patients had a higher hip COR. In Kong et al.'s study, all patients experienced an average upward COR migration of 3.7 mm postoperatively, with average absolute anterior-posterior and mediolateral displacements of 4.1 and 2.7 mm, respectively (Kong et al., 2022). These migrations may result from displacement between the augment and the cup, impacting prosthesis stability. In our study, up to the most recent follow-up, no prosthesis migration was observed in any patient.

Integrated implants and personalized surgical planning can effectively reduce operative time. Compared to modular implants, which require step-by-step installation, fixation, and verification of each component, integrated implants offer ease of installation, saving time and minimizing intraoperative bleeding. Hu et al. (2024) utilized 3D printing technology to customize implants and conducted preoperative planning, achieving an average operative time of 275.1 ± 94.0 min and an average intraoperative blood loss of 1896.9 ± 801.1 mL. In Wang et al. (2020) study on hemipelvic prosthesis reconstruction, the median blood loss was 2,600 mL (ranging from 900 to 8,200 mL). Although our patients routinely received postoperative blood transfusions, their intraoperative blood loss was less than that reported in previous studies. This further underscores the advantage of integrated implants in simplifying surgical procedures.

This study had several limitations. Firstly, the sample size of clinical case series was relatively small, and secondly, there was a lack of control group. This was mainly due to the difficulty to obtain a sufficient source of patients of complex periacetabular bone defects that meet the indications, as such patients themselves were relatively scarce. Moreover, the surgical procedure of complex periacetabular bone defects is challenging. Even for experienced orthopedic surgeons, it is very difficult to carry out large-scale new surgical technologies in treating complex periacetabular bone defects. Therefore, based on previous research (Wang et al., 2024), the primary objective of this study was to further comprehensively present the application advantages of TC4-based tantalum-coated prostheses and introduce our innovative concept through *in vivo* osteogenic experiments using animal models and detailed elaboration of clinical applications. Meanwhile, as the technology gradually advances, we will keep expanding the clinical sample size and prolonging the follow-up period for further evaluation of the long-term clinical efficacy and related complications.

## 5 Conclusion

The purpose of this study was to introduce the 3D printed TC4-based Ta-coated prostheses for treating complex periacetabular bone defects. *In vivo* experiments in minipigs demonstrated that the tantalum (Ta) coating improved early-stage osseointegration compared to uncoated TC4 scaffolds, along with good local biocompatibility. Meanwhile, in a clinical case series of 3 patients with complex acetabular bone defects, the TC4-based Ta-coated prosthesis showed preliminary clinical benefits, including significant relief of pain symptoms, improvement in Harris Hip Score (HHS), and no severe complications during an average follow-up of 27.7 months. These results suggested that the Ta coating may contribute to enhanced osseointegration of TC4-based prostheses, providing a potential alternative for the reconstruction of complex acetabular bone defects. However, further validation with larger sample sizes and longer-term follow-up is required to confirm its long-term safety and efficacy.

## Data Availability

The original contributions presented in the study are included in the article/supplementary material, further inquiries can be directed to the corresponding author.
